# Persistently high burden of acute respiratory infections requiring hospitalization in German pediatric hospitals, fall/winter 2023–2024

**DOI:** 10.1007/s15010-025-02494-z

**Published:** 2025-03-11

**Authors:** Svenja Dreßen, Josephine Schneider, Maren Doenhardt, Natalie Diffloth, Tobias Tenenbaum, Dominik T. Schneider, Andreas Trotter, Nicole Toepfner, Reinhard Berner, the DGPI Ad hoc-ARI-register working group, Svenja Dreßen, Josephine Schneider, Tobias Ankermann, Felicitas Anselmino, Stefan Arens, Sven Armbrust, Volker Arpe, Elisabeth Bach, Gerald Beier, Christiane Bell, Martin Berghäuser, Malte Bergmann, Sonja Bernlochner, Alexander Bey, Anke Beyersdorff, Julia Blumör, Jasmin Brühler, Reinhard Bullmann, Reimer Conzelmann, Roland Degener, Catharina Dobberschütz, Thomas Eckardt, Torsten Ehrchen, Christoph Ehrsam, Matthias Endmann, Florian Epple, Michael Fedlmeier, Holger Freymann, Marianne Funken, Marie Gah, Viola Gerstmann, Christine Goletz, Tatiana Görhardt, Katrin Gröger, Julia Handermann, Madeleine Haug, Solvej Heidtmann, Maik Heine, Matthias Henschen, Alexander Herz, Mirjam Höfgen, Daniel Hubert, Birte Hunfeld, Conny Huster, Katja Hüwe, Kristin Jähnert, Daniela Jerzyk, Petra Kaiser-Labusch, Marcus Kania, Jens Kästner, Margit Kellerer, Svetlana Kelzon, Chrisoph Kemen, Matthias Kettwig, Alexander Kiefer, Karoline Kinkelin, Andreas Klein, Christoph Klein, Anna-Lisa Kleinmaier, Katharina Kliemann, Jan Knechtel, Felix Knirsch, Louise Kobelt, Barbara Korinth, Ekaterini Kougioumtzi, Georgia Koukli, Benno Kretzschmar, Johannes Kugler, Miriam Landauer, Alfred Längler, Tina Liebscher, Vanessa Lionetti, Daniela Lubitz, Anna-Lena Lubojanski, Sa Luo, Julia Lutsch, Friederike Mattay, Anja Mayer, Miriam Meusel, Ulf Meyer, Marko Mohorovicic, Johanna Mösler-Tuczek, Annika Müller, Sven Nipken, Ursula Pindur, Erika Plattner, Simone Pötzsch, Lutz Pross, Andreas Rößlein, Meike Rumpf, Jörn Schamell, Norbert Schmeja, Juliane Schmid, Dominik Schneider, Julia Schönherr, Peter Schonhoff, Maria Sichardt, Daniel Steer, Detlef Stein, Frank Stemberg, Julia Tabatabai, Norbert Teig, Wolfgang Thomas, Renate Turan, Vanda Tuxhorn, Mirjam Ungerechts, Christian v. Schnakenburg, Alijda Ingeborg van den Heuvel, Noemi Vereb, Simone Wagner, Sarah Wiehl, Christiane Maria Wiethoff, Elisabeth Wittig, Anne Zeller, Ulrich Zügge

**Affiliations:** 1 Department of Pediatrics, University Hospital and Medical Faculty Carl Gustav Carus, Technische Universität Dresden, Fetscherstrasse 74, 01307 Dresden, Germany; 2 Department of Pediatrics and Adolescent Medicine, Paracelsus Medical University, University Hospital Salzburg, Salzburg, Austria; 3 Clinic for Child and Adolescent Medicine, Sana Klinikum Lichtenberg, Academic Teaching Hospital, Charité-Universitätsmedizin Berlin, Berlin, Germany; 4 Clinic of Pediatrics, Municipal Hospital Dortmund, University Witten/Herdecke, Dortmund, Germany; 5 Children’s Hospital and Center for Perinatal Medicine, Teaching Hospital of the University of Freiburg, Singen, Germany

**Keywords:** Acute respiratory infection, RSV, Influenza, SARS-CoV-2, Children, Hospitalization

## Abstract

**Purpose:**

During fall 2021, children’s hospitals in Germany faced a surge in RSV-related hospitalizations, whereas during fall/winter 2022–2023, RSV and influenza infections both led to increased inpatient admissions. Our study prospectively assessed severe acute respiratory infections, their causative pathogens, and the resulting disease burden on German children's hospitals for the fall/winter 2023–2024 season.

**Methods:**

From October 3, 2023 through April 16, 2024, children hospitalized with ARI as a primary diagnosis were monitored via a national survey established by the German Society for Pediatric Infectious Diseases (DGPI). Weekly data was collected on total hospital admissions, ARI-related admissions by pathogen (SARS-CoV-2, RSV, influenza, other), ICU admissions with ARI as a primary diagnosis, and respiratory support.

**Results:**

Overall, 23% of German children's hospitals (77/334 centers) submitted 1234 survey reports. ARI-related hospital admissions surged starting in November 2023 and peaked in late December 2023 (53.4% of all admissions), in parallel with a peak in the average number of newly-admitted patients (aNA) with RSV (2.5 aNA). In comparison to the 2022/2023 season, fewer newborns and infants were admitted for ARI (4.7%, p < 0.001/1.9%, p = 0.05) and fewer required ICU treatment (5.3%, p = 0.02/5.6%, p = 0.001 respectively). In 74.9% of ICU patients, ventilation support was required—9.1% less than in the previous season.

**Conclusion:**

The clinical burden on pediatric hospitals and ICUs is strongly influenced by the changing, annually circulating pathogens and affected age group. Therefore, a continuous, systematic, dynamic collection of ARI data is critical for assessing the ARI-related morbidity and the associated burden on health care systems.

**Supplementary Information:**

The online version contains supplementary material available at 10.1007/s15010-025-02494-z.

## Introduction

The Ad-hoc ARI Survey of the German Society for Pediatric Infectious Diseases (DGPI) was launched in 2021 to track the intra- and post-pandemic development of infectious diseases among children and adolescents. The survey’s goal was to analyze seasonal patterns of respiratory pathogens and the burden on children's hospitals in Germany during the fall/winter seasons. The focus was to capture: (a) total number of pediatric hospital admissions (regardless of admission diagnosis); (b) number of patients admitted with acute respiratory infections (ARI) as the primary reason for admission; and (c) diagnoses with respiratory viruses (especially RSV, influenza and SARS-CoV-2) that were able to be detected in those ARI patients. Following the data collection, we compared the 2022–2023 results to surveillance data from fall/winter 2021–2022. Our previous analysis had shown that seasonal patterns of infectious diseases shifted toward the conclusion of the pandemic [1], with SARS-CoV-2 infections steadily declining and RSV infections sharply increasing [2].

## Materials and methods

The survey was promoted via the websites of the DGPI and the German Society of Pediatrics (DGKJ), and additionally was announced in the DGPI and DGKJ newsletters. From October 3, 2023, to April 16, 2024, German hospitals submitted anonymized data on hospital admissions of children and adolescents who had ARI as their main diagnosis. Data on numbers and diagnoses of pediatric hospital admissions were captured every Tuesday (as the weekly reference day) by voluntary report. Data collected were publicly accessible via the DGPI website [3]. Captured data included: (1) whether admission primarily was due to ARI or for another reason; (2) type of respiratory pathogens leading to hospital admission and/or intensive care (ICU) treatment; and (3) patient age group. The main ARI pathogens were: SARS-CoV-2, RSV, and influenza virus. Survey participants additionally had the option of choosing "other confirmed respiratory pathogen". In cases where pathogen detection was pending or missing, the survey allowed participants to select "unknown pathogen". For ICU patients, the survey collected additional data regarding need for respiratory support (invasive vs. non-invasive) and whether or not patients had significant comorbidities.

The survey additionally assessed coinfections. Guidelines for survey participants stated the following reporting procedures: When calculating total number of patients (new admissions or admissions to ICU), patients with coinfections were to be counted only once. In the pathogen-specific part of the survey, documentation of coinfections was requested, including each of the relevant pathogens detected.

Age groups were defined as: newborns (0–3 months of life), infants (4–11 months), toddlers (1–2 years), preschool-aged children (3–4 years), school-aged children (5–11 years), adolescents (12–18 years) and young adults (≥ 19 years).

Study data were collected and managed using REDCap (Research Electronic Data Capture) software, hosted at the Technical University Dresden, Germany [4, 5]. Analyses were performed using Microsoft Excel v.2010. Graphs were created using Datawrapper software (datawrapper.de). Descriptive statistics were presented as absolute frequencies with percentages for categorical variables. To prevent reporting bias due to different participation rates during the study period, average case numbers were calculated based on cases reported per reporting hospital per day. They were additionally coded as average numbers of newly-admitted patients per reporting hospital per day (aNA) and as average numbers of ICU patients who had ARI as a main diagnosis per reporting hospital per day (aICU). When calculating the aICU, only hospitals with an in-house ICU were included. All data sets were checked by working group members. In consultation with the data collectors, plausibility checks were carried out and missing data supplemented or justified. Finally, results were compared to the survey data from fall/winter 2022–2023 [1]. P-values were calculated using Fisher’s exact-Test with a significance level of 0.05.

The survey was reviewed and approved by the Ethics Committee of the Technical University Dresden.

## Results

### Dynamic proportions of different respiratory pathogens over time

Over the full timeframe of the survey, 1234 reports from 77 children's hospitals were submitted—a participation rate equivalent to 23.1% of all German children's hospitals, including both small and large hospitals and both primary and tertiary care. At the peak of survey participation (January 16, 2024), 59 of 334 German pediatric hospitals (17.7%) submitted reports, of which 44 had ICU departments (74.6%). The lowest participation rate was during the initial survey period (October 10, 2023), when 5 (1.5%) hospitals participated, of which 2 had ICU departments (40%). Over the full study period, the median number of reporting hospitals was 43 (12.7%).

In total, 3014 newly-admitted patients and 921 ICU patients with ARI as their main diagnosis were reported (Table 1). An average of 396 patients per weekly reference day (Tuesdays) were hospitalized, with a peak of 602 absolute cases per day occurring on November 28, 2023. At 53% of total admissions (3.7 of 6.9 aNA), ARI admissions reached their peak on December 26, 2023. Shortly afterwards, the proportion of ARI and aNA decreased, with just 21.6% (2.0 aNA) being recorded on January 9, 2024. After a second increase in ARI admissions at the end of January 2024 (35.2%; 3.3 of 9.5 aNA), the rate remained < 25% after February 20, 2024, while the aNA varied between 1.2 and 2.1.

**Table 1:** Absolute case numbers of patients reported for the fall/ winter seasons of 2022–2023 vs. 2023–2024: (a) newly-admitted patients with an acute respiratory infection (ARI) as their primary admission diagnosis; (b) ICU patients with ARI as a primary diagnosis

	All			SARS-CoV-2			RSV		
	2022–2023	2023–2024	p-value	2022–2023	2023–2024	p-value	2022–2023	2023–2024	p-value
	N (%)	N (%)	***	N (%)	N (%)	***	N (%)	N (%)	***
Patients									
(a) newly- admitted	3325	3079		252 (7.6)	203 (6.6)	0.13	1005 (30.2)	1008 (32.7)	0.03
Age									
Newborns	745 (22.4)	546 (17.7)	< 0.001	94 (37.3)	84 (41.4)	0.39	423 (42.8)	295 (29.3)	< 0.001
(0–3 months)									
Infants (4–11 months)	612 (18.4)	626 (20.3)	0.05	65 (25.8)	61 (30.0)	0.34	277 (27.6)	290 (28.8)	0.55
Toddlers (1–2 years)	857 (25.8)	820 (26.6)	0.44	40 (15.9)	27 (13.3)	0.51	187 (18.6)	275 (27.3)	< 0.001
Preschool-aged children (3–4 years)	485 (14.6)	443 (14.4)	0.83	10 (4.0)	9 (4.4)	0.82	75 (7.4)	112 (11.1)	0.01
School-aged children (5–11 years)	424 (12.8)	418 (13.6)	0.34	22 (8.7)	10 (4.9)	0.14	22 (2.2)	27 (2.7)	0.56
Adolescents	123 (3.7)	178 (5.8)	< 0.001	21 (8.3)	12 (5.9)	0.37	7 (0.7)	4 (0.4)	0.39
(12–18 years)									
Young adults	2 (0.1)	2 (0.1)	1.0	1 (0.4)	0 (0.0)	1.0	0 (0.0)	0 (0.0)	1.0
(≥ 19 years)									
Age not specified	78 (2.3)	46 (1.5)	0.01	0 (0.0)	0 (0.0)	1.0	14 (1.4)	5 (0.5)	0.04
(b) on intensive care unit (ICU)	795	921		34 (4.3)	35 (3.8)	0.62	328 (41.3)	392 (42.6)	0.59
Age									
Newborns	265 (33.3)	258 (28.0)	0.02	8 (23.5)	10 (28.6)	0.79	192 (58.5)	201 (51.3)	0.06
(0–3 months)									
Infants (4–11 months)	143 (18.0)	114 (12.4)	0.001	14 (41.2)	9 (25.7)	0.21	65 (19.8)	59 (15.1)	0.09
Toddlers (1–2 years)	147 (18.5)	172 (18.7)	0.95	1 (2.9)	11 (31.4)	0.003	37 (11.3)	67 (17.1)	0.03
Preschool-aged children (3–4 years)	69 (8.7)	116 (12.6)	0.01	2 (5.9)	2 (5.7)	1.0	13 (4.0)	26 (6.6)	0.14
School-aged children (5–11 years)	99 (12.5)	164 (17.8)	0.003	3 (8.8)	3 (8.6)	1.0	7 (2.1)	32 (8.2)	< 0.001
Adolescents	59 (7.4)	82 (8.9)	0.29	5 (14.7)	0 (0.0)	0.02	10 (3.1)	7 (1.8)	0.33
(12–18 years)									
Young adults	3 (0.4)	1 (0.1)	0.34	2 (5.9)	0 (0.0)	0.24	1 (0.3)	0 (0.0)	0.46
(≥ 19 years)									
Age not specified	11 (1.4)	14 (1.5)	0.84	0 (0.0)	0 (0.0)	1.0	3 (0.9)	0 (0.0)	0.09
On ICU, including additional information on comorbidities**	527 (66.3)	609 (66.1)	0.96	28 (82.4)	30 (85.7)	0.75	186 (56.7)	208 (53.1)	0.33
On ICU with comorbidities**	268 (50.9)	398 (65.4)	< 0.001	15 (53.6)	19 (63.3)	0.59	63 (33.9)	109 (52.4)	< 0.001
On ICU, including additional information on respiratory support**	698 (87.8)	856 (92.9)	< 0.001	30 (88.2)	31 (88.6)	1.0	289 (88.1)	365 (93.1)	0.03
On ICU with respiratory support**	586 (84.0)	641 (74.9)	< 0.001	19 (63.3)	25 (80.6)	0.16	265 (91.7)	277 (75.9)	< 0.001
With invasive ventilation**	131 (18.8)	194 (22.7)	0.07	9 (30.0)	11 (35.5)	0.79	34 (11.8)	64 (17.5)	0.05
With non-invasive ventilation**	455 (65.2)	447 (52.2)	< 0.001	10 (33.3)	14 (45.2)	0.43	231 (79.9)	213 (58.4)	< 0.001

	Influenza			other confirmed respiratory pathogens			unknown respiratory pathogens*		
	2022–2023	2023–2024	p-value	2022–2023	2023–2024	p-value	2022–2023	2023–2024	p-value
	N (%)	N (%)	***	N (%)	N (%)	***	N (%)	N (%)	***
Patients									
(a) newly- admitted	444 (13.4)	427 (13.9)	0.56	286 (8.6)	187 (6.1)	< 0.001	1338 (40.2)	1254 (40.7)	0.70
Age									
Newborns	50 (11.3)	36 (8.4)	0.17	30 (10.5)	17 (9.1)	0.64	148 (11.1)	114 (9.1)	0.1
(0–3 months)									
Infants (4–11 months)	47 (10.6)	59 (13.8)	0.15	38 (13.3)	33 (17.6)	0.24	185 (13.8)	183 (14.6)	0.61
Toddlers (1–2 years)	92 (20.7)	98 (23.0)	0.46	88 (30.8)	48 (25.7)	0.25	450 (33.6)	372 (29.7)	0.03
Preschool-aged children (3–4 years)	95 (21.4)	84 (19.7)	0.56	66 (23.1)	30 (16.0)	0.08	239 (17.9)	208 (16.6)	0.41
School-aged children (5–11 years)	115 (25.9)	117 (27.4)	0.65	46 (16.1)	31 (16.6)	0.89	219 (16.4)	233 (18.6)	0.15
Adolescents	38 (8.6)	31 (7.3)	0.53	12 (4.2)	22 (11.8)	0.003	45 (3.4)	109 (8.7)	< 0.001
(12–18 years)									
Young adults	0 (0.0)	1 (0.2)	0.49	0 (0.0)	0 (0.0)	1.0	1 (0.1)	1 (0.1)	1.0
(≥ 19 years)									
Age not specified	7 (1.6)	1 (0.2)	0.07	6 (2.1)	6 (3.2)	0.55	51 (3.8)	34 (2.7)	0.12
(b) on intensive care unit (ICU)	105 (13.2)	128 (13.9)	0.72	183 (23.0)	220 (23.9)	0.69	145 (18.2)	146 (15.9)	0.2
Age									
Newborns	14 (13.3)	6 (4.7)	0.03	28 (15.3)	22 (10.0)	0.13	23 (15.9)	19 (13.0)	0.51
(0–3 months)									
Infants (4–11 months)	11 (10.5)	10 (7.8)	0.5	35 (19.1)	21 (9.5)	0.01	18 (12.4)	15 (10.3)	0.58
Toddlers (1–2 years)	26 (24.8)	23 (18.0)	0.26	50 (27.3)	39 (17.7)	0.02	33 (22.8)	32 (21.9)	0.89
Preschool-aged children (3–4 years)	17 (16.2)	22 (17.2)	0.86	20 (10.9)	47 (21.4)	0.01	17 (11.7)	19 (13.0)	0.86
School-aged children (5–11 years)	24 (22.9)	47 (36.7)	0.02	30 (16.4)	56 (25.5)	0.03	35 (24.1)	26 (17.8)	0.2
Adolescents	11 (10.5)	20 (15.6)	0.33	18 (9.8)	30 (13.6)	0.28	15 (10.3)	25 (17.1)	0.12
(12–18 years)									
Young adults	0 (0.0)	0 (0.0)	1.0	0 (0.0)	0 (0.0)	1.0	0 (0.0)	1 (0.7)	1.0
(≥ 19 years)									
Age not specified	2 (1.9)	0 (0.0)	0.2	2 (1.1)	5 (2.3)	0.46	4 (2.8)	9 (6.2)	0.26
On ICU, including additional information on comorbidities**	71 (67.6)	99 (77.3)	0.1	130 (71.0)	167 (75.9)	0.31	112 (77.2)	105 (71.9)	0.35
On ICU with comorbidities**	44 (62.0)	78 (78.8)	0.02	75 (57.7)	122 (73.1)	0.01	71 (63.4)	70 (66.7)	0.67
On ICU, including additional information on respiratory support**	89 (84.8)	121 (94.5)	0.02	159 (86.9)	211 (95.9)	0.002	131 (90.3)	128 (87.7)	0.57
On ICU with respiratory support**	67 (75.3)	95 (78.5)	0.62	133 (83.6)	150 (71.1)	0.01	102 (77.9)	94 (73.4)	0.47
With invasive ventilation**	22 (24.7)	42 (34.7)	0.13	42 (26.4)	59 (28.0)	0.81	24 (18.3)	18 (14.1)	0.4
With non-invasive ventilation**	45 (50.6)	53 (43.8)	0.4	91 (57.2)	91 (43.1)	0.01	78 (59.5)	76 (59.4)	1.0

Age distribution is shown for each pathogen. For ICU patients, absolute numbers for those with comorbidities and/or with need for respiratory support are stated for different pathogen groups only in cases where information was submitted. (Entry of this additional information was optional.)

^*^Pathogen detection pending/not performed

**Percentages are calculated in relation to absolute case numbers in reports where information was provided

***Statistical analysis was performed with the Fisher’s exact-Test

**Fig. 1:**
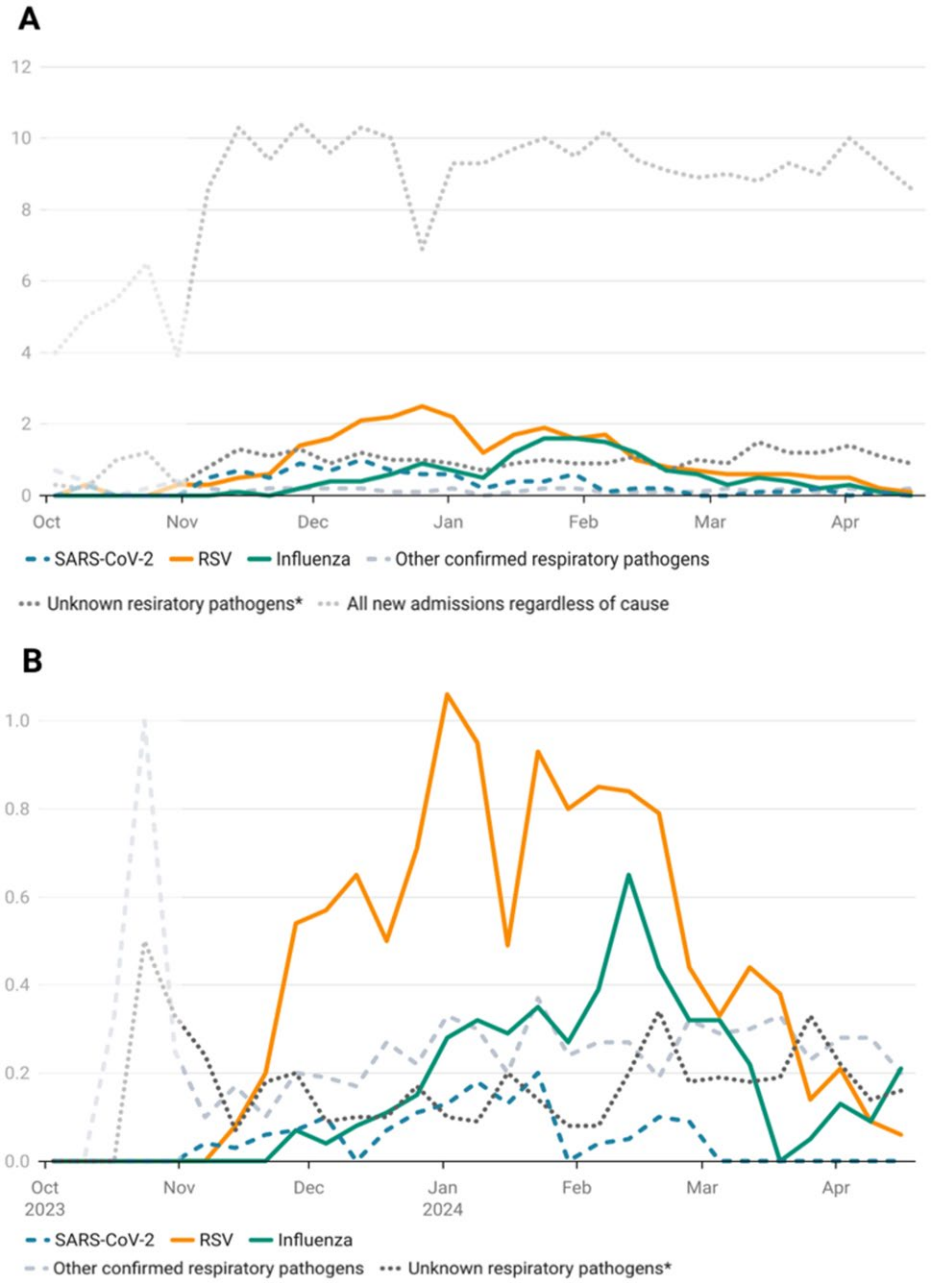
**A** New hospital admissions with respiratory tract infections, average cases per day per reporting hospital. **B** Number of patients receiving ICU treatment due to a respiratory tract infection, average cases per day per reporting hospital with an in-house ICU. *Pathogen detection pending/not performed

Over the course of the full survey period, a median of 2.2 ARI-related admissions per reporting hospital per day was recorded (range 0.6–3.7 aNA), and a median of 0.8 patients (range 0.0–1.5 aICU) received ARI-related ICU treatment (Fig. 1B). One week after the first aNA peak (December 26, 2023) and two weeks after the second aNA peak (January 30, 2024), average ARI cases on ICU peaked at 1.4 aICU.

With a median of 0.3 aNA (range 0.0–1.0) and 0.05 aICU (range 0.0–0.2), SARS-CoV-2 played only a minor role compared to other respiratory pathogens. Of note, however, in December 2023, there was a slight increase in SARS-CoV-2-related admissions and ICU hospitalizations (1.0 aNA on December 12, and 0.2 aICU on January 23, 2024) (Fig. 1).

Other commonly-reported pathogens included rhino-/enterovirus (n = 50, 27%), *Mycoplasma pneumoniae* (n = 38, 21%), adenovirus (n = 30, 16%) and parainfluenzavirus (n = 18, 10%), as well as smaller numbers of other pathogens, including *Streptococcus pneumoniae*, *Streptococcus pyogenes*, *Haemophilus influenzae*,* Staphylococcus aureus*, *Pseudomonas aeruginosa,* and *Klebsiella pneumoniae*. Coinfections occurred in 8.5% of newly-admitted patients (n = 81 of all reports) and in 17.6% of ICU patients (n = 74 of all reports). Unknown pathogens (e.g., cases with pathogen detection pending or not performed) were reported in 41.8% (n = 1432) of all newly-admitted patients with ARI as their primary diagnosis for hospital admission.

### Age distribution and respiratory support: Comparison of the fall/winter 2022–2023 and 2023–2024 seasons

Compared to the previous season (Table 1), there was a shift in the age distribution of ARI-related hospitalizations in fall/winter 2023–2024. Overall, newborns were admitted less frequently due to ARI (n = 546/3079 vs. n = 745/3325, – 4.7%, p < 0.001) and also were less frequently treated on ICU due to ARI (n = 258/921 vs. n = 265/795, – 5.3%, p = 0.02). The number of adolescents as a percentage of total admissions increased by 2.1% (n = 178/3079 vs. n = 123/3325, p < 0.001). 5.3% more school-aged children were treated on ICU than during the previous year (n = 164/921 vs. n = 99/795, p = 0.003). This shift is particularly evident when evaluating RSV and influenza infections. The total number of newly-admitted RSV patients increased by 2.5% (n = 1008/3079 vs. n = 1005/3325, p = 0.03), while the number of RSV-patients on ICU remained stable (42.6% vs. 41.3%). A closer look at the age groups does, however, reveal differences in the frequency of admissions to ICU due to RSV. Compared to the previous year, 1–2-year olds were affected 5.8% more frequently (n = 37/328 vs. n = 67/392, p = 0.03) and school-aged children 6.1% (n = 7/328 vs. n = 32/392, p < 0.001). Due to influenza, 8.6% fewer newborns were admitted to ICU than in the previous year (n = 14/105 vs. n = 6/128, p = 0.03), while 13.8% more school-aged children required intensive care treatment (n = 24/105 vs. n = 47/128, p = 0.02). The proportion of other confirmed respiratory pathogen-related admissions among adolescents increased by 7.6% over the previous year (n = 22/187 vs. n = 12/286, p = 0.003). While the number of intensive care admissions due to other confirmed respiratory pathogens decreased 9.6% for both infants (n = 35/183 vs. n = 21/220, p = 0.01) and toddlers (n = 50/183 vs. n = 39/220, p = 0.02), it increased 10.5% for preschool children (n = 20/183 vs. n = 47/220, p = 0.01) and 9.1% for school-aged children (n = 30/183 vs. n = 56/220, p = 0.03).

Of 921 patients admitted to the ICU, additional information on respiratory support was provided in 856 reports. In 74.9% of these patients (n = 641/856), ventilation support was provided – 9.1% less than during the previous year (n = 586/698, p < 0.001). Among RSV patients on ICU for whom information on respiratory support was available, 75.9% required respiratory support (n = 277/365), which was 15.8% less than during the previous year (n = 265/289, p < 0.001). The majority (58.4%, n = 213/365) required non-invasive ventilation, including CPAP or high flow oxygen*.* After SARS-CoV-2 patients, influenza patients represented second highest proportion of those receiving invasive ventilation (n = 42/121, 34.7%).

### Comparison of average RSV and Influenza cases per day per reporting hospital, fall/winter seasons 2021–2022 vs. 2022–2023 vs. 2023–2024

In 2021, the highest incidence of RSV-related admissions was reported at the end of October/beginning of November (3.0 RSV aNA on 19 October, 2021), with numbers sharply dropping by mid-December. Unfortunately, no data on influenza were collected for the fall/winter 2021–2022 season. During the 2022–2023 season, peaks in RSV and influenza cases (3.6 RSV aNA on December 6, 2022, and 2.1 influenza aNA on December 13, 2022) coincided with the peak in reported ARI admissions. Two weeks after the aNA peak (December 20, 2022), the average number of ARI cases on ICU peaked at 2.9 aICU, which is also when RSV (1.6 aICU) and influenza cases (1.2 aICU) reached their highest point. During the 2022–2023 season, the number of patients admitted to ICU for RSV infections nearly doubled over those of the previous year. While RSV and influenza admissions declined after mid-January 2023, a slight increase in influenza-related ICU hospitalizations (0.7 aNA) was observed at the end of March 2023 (0.2 aICU, March 21).

During the 2023–2024 season, the peaks in RSV and influenza cases (2.5 RSV aNA on December 26, 2023, and 1.6 influenza aNA on January 30, 2024) again coincided with the peaks in reported ARI admissions, as was the case the year before. However, the highest rates of RSV and influenza ICU hospitalization occurred at different times, with RSV peaking in early January (1.1 aICU) and influenza peaking in mid-February (0.7 aICU). Following these peaks, RSV cases declined rapidly, while influenza-related ICU hospitalizations remained stable at a low level until mid-March (0.2 aICU, March 12, 2024).

## Discussion

During fall/winter seasons, overcrowding in emergency departments of children's hospitals in Europe has been a persistent problem. With 23.1% of German pediatric hospitals contributing data to our survey in the 2023–2024 season, our study presents a unique approach for estimating the disease burden of ARI-related hospitalizations and intensive care. Encouragingly, the average participation of reporting pediatric departments slightly increased between the 2022–2023 and 2023–2024 seasons. This underlines a growing awareness for the importance of continuous surveillance of infectious diseases among children, and also suggests confidence in our survey. Overall, the burden of respiratory infections during fall/winter 2023–2024 remained unchanged compared to the previous year (2022–2023: 2.6 aNA and 0.8 nICU vs. 2023–2024: 2.2 aNA and 0.8 aICU).

**Fig. 2:**
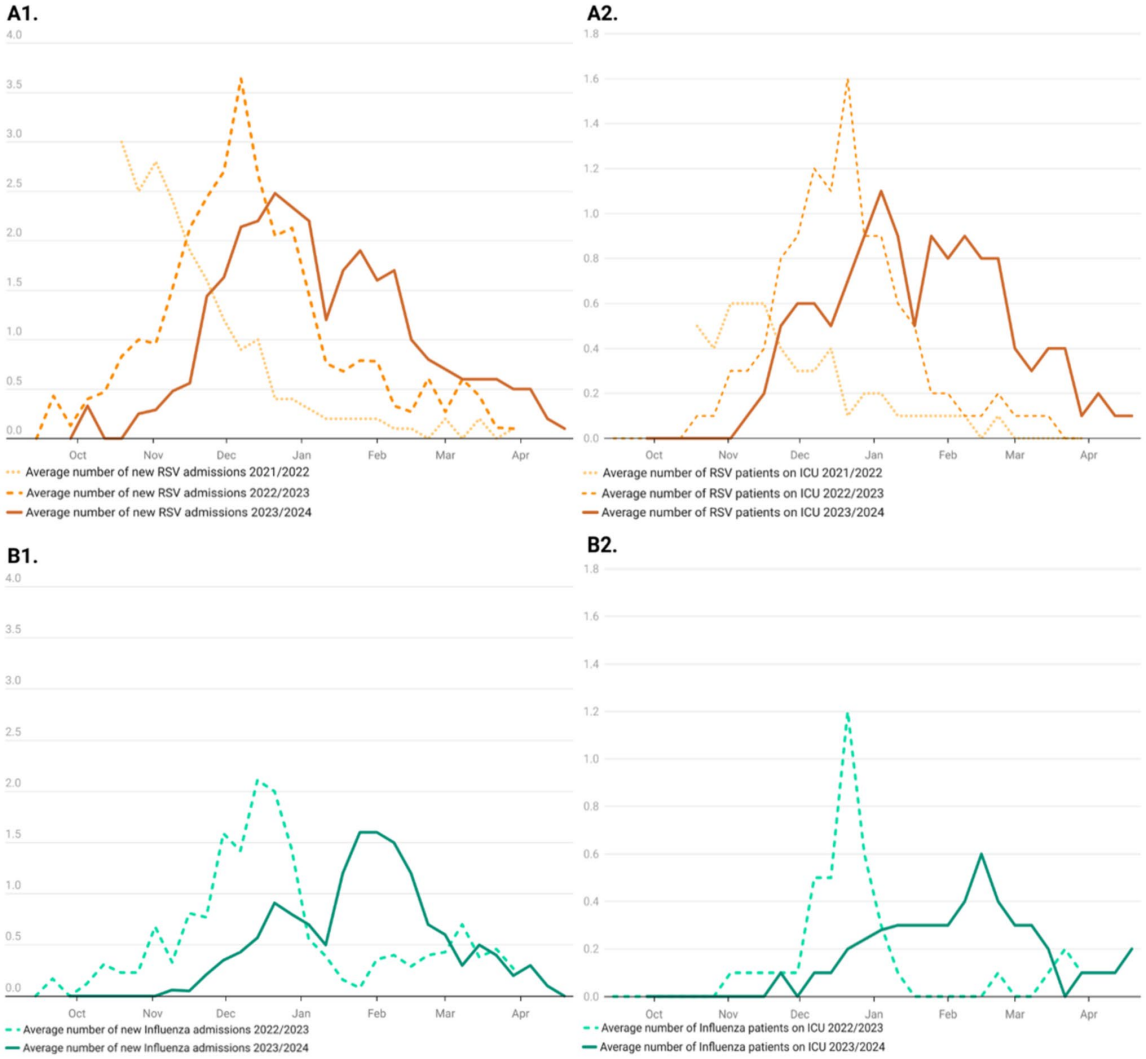
**A1** Average number of new RSV admissions, average cases per day per reporting hospital. **A2** Average number of RSV patients receiving ICU treatment, average cases per day per reporting hospital with an in-house ICU. **B1** Average number of new Influenza admissions, average cases per day per reporting hospital. **B2** Average number of Influenza patients receiving ICU treatment, average cases per day per reporting hospital with an in-house ICU. As there was no calendar week 53 at the end of 2023, average value was calculated for illustrative purposes only

Launched in October 2021, our survey enables the capture of data regarding the changing seasonal patterns of RSV during the post-pandemic era. While in previous years, the RSV season in Germany usually peaked in February/March [6], our 2023–2024 data shows there to have been a significant shift in the timing of its seasonal peak. While RSV peaked unusually early in November 2021 [7], in 2022, it shifted to the beginning of December [1], and in 2023 to the end of December. This suggests that the timing of RSV’s seasonality slowly returned to pre-pandemic patterns as the pandemic drew to a close (Fig. 2).

Among influenza infections, a similar development – a return to pre-pandemic patterns – can be seen. During the 2023–2024 season, the peak of influenza infections shifted to the end of January 2024, while the high burden of hospital admissions lasted until mid-March 2024 (Fig. 2). In December 2022, an especially early influenza wave, one occurring in parallel with a RSV wave, particularly burdened children's hospitals in Germany [8]. During the 2023–2024 season, a more prolonged two-peaks ARI pattern developed.

Pathogen distribution by age group during the 2023–2024 season shifted only slightly. Newborns and infants were less affected overall, while school-aged children and adolescents were more frequently hospitalized, particularly when patients were admitted with other confirmed respiratory pathogens. In this season, RSV and SARS-CoV-2 primarily affected newborns and infants, while influenza was more common among preschool and school children. Comorbidity rates remained stable at approximately 50% (Table 1).

Although SARS-CoV-2 did not represent a significant disease burden for children and adolescents in Germany during the fall/winter 2022–2023 season [1], RSV, influenza, and other ARI pathogens continued to challenge the capacity of the pediatric hospital care system.

In addition to rhinoviruses and enteroviruses (27%), *Mycoplasma pneumoniae* was reported in 21% of those diagnosed with “other respiratory pathogens”. Although this diagnosis category was not separately tracked by our survey, the number suggests an increased prevalence of *M. pneumoniae* infections during the past fall/winter season and is in line with other international research findings [3, 9, 10].

While 84% of ARI-patients treated on ICU received respiratory support (including CPAP and high-flow oxygen) during the 2022–2023 season, the percentage fell to 74.5% in the following season. The decline of respiratory support for patients with RSV infections is particularly noteworthy: here the proportion fell by 15.8% (p < 0.001). Although the number of SARS-CoV-2 cases was low overall (6.6%), newborns and infants (71.4%) were particularly at risk. During the 2023–2024 season, 80.6% of ICU patients were on respiratory support, representing the largest proportion. Influenza patients also were frequently affected (78.5%) and had a higher proportion of invasive ventilation (34.7%). At a rate of approximately half, the proportion of patients with comorbidities remained similar across both seasons.

The high number of influenza-related admissions to ICUs during the 2023–2024 season is especially striking. Typically, influenza infections do not lead to ICU admissions. We hypothesize that the post-pandemic increase in influenza infections may have been related to reduced immunity, since contact restrictions during the pandemic likely lowered overall antigen exposure in children. In addition, newborns and infants are known to be a particularly vulnerable group. Otherwise, however, the reason for the increased number of ICU admissions appears unclear.

Our study emphasizes the importance of monitoring ARI among children in order to assess the overall disease burden in a given country or region. This is particularly true when the burden of peak acute hospitalizations related to seasonality of pediatric ARI poses a challenge to pediatric departments, whether in emergency rooms, regular wards or on ICU. However, despite its value, our survey design has limitations. The survey is based on voluntary participation and had varying reporting rates. Although one quarter of pediatric departments in Germany reported data, this nevertheless could result in bias. A mandatory and centralized monitoring system might overcome such challenges. The specific methods of pathogen detection were not queried in this study. Due to established and improved standard procedures in pathogen diagnostics in German pediatric departments in recent years, it can be assumed that the results are comparable. Nevertheless, the weekly ad hoc survey was primarily aimed at rapid pathogen detection, which by nature is PCR- and antigen test-based, especially with regard to viral pathogens. It is possible that bacterial infections were underreported. Due to the study design, we were unable to evaluate coinfections in detail, but our survey shows that they exist and may play a role in prolonging hospitalization, especially among infants, toddlers and children with underlying disease. A standardization of collection methods and resource data acquisition also would improve data quality and enable a more comprehensive, reliable surveillance of ARI and other infectious diseases among children. However, this requires resources and public funding. Furthermore, the primary aim of this survey was to monitor the general ARI-related disease burden on children's hospitals in Germany by collecting anonymized data on ARI-related hospitalizations. Due to the nature of such a survey, we were unable to collect information about, or draw any conclusions regarding, individual clinical courses, risk factors, or vaccination status. By requesting that survey participants report the main diagnoses on admission, our survey captured the burden of hospitalizations for ARI, while also minimizing the risk of incidental positive test results.

In summary, during the fall/winter of 2023–2024 there was a slight decrease in the burden of respiratory infections as compared to the previous year. RSV and influenza infections continued to account for the largest proportion of reported cases. RSV infections increased in mid-October and peaked at the end of December. This was followed by a sharp rise in influenza cases in January/February 2024, with high rates of ICU admissions and continued high rates of RSV infection. In absolute numbers, SARS-CoV-2 continued to play a minor role during the 2023–2024 season, but those most severely affected were newborns and infants.

## Conclusion

Overall, current trends point toward a return to pre-pandemic seasonal infection patterns for ARI in Germany. Our ability to report such trends emphasize the importance of continuous, dynamic, systematic surveillance of ARI data (as well as of other infectious diseases) in order to monitor actual burdens on hospital-based healthcare systems. Our survey affirms the value of weekly ad hoc-surveys as a tool for the real-time monitoring and evaluation of pediatric hospital care at the national level. Such surveys also can help inform future allocation of healthcare resources. The high voluntary participation rates our survey achieved among hospitals, as well as the high quality of data submitted, are critical components for any surveillance tool. In the future, effective surveillance of respiratory diseases requires standardized data collection, integrated data sources, real-time data analysis and early warning systems. Incorporating outpatient data and promoting public involvement enhance an understanding of the overall infection landscape. Advancements in diagnostics, mathematical modeling and artificial intelligence (AI) are expected to be crucial for future surveillance. These would enable hospitals to preserve staff resources and ensure even more comprehensive monitoring of ARI epidemiology in children, which in turn would offer substantial benefits to the health care system. It additionally would benefit future pandemic preparedness as recommended by the WHO.

## Supplementary Information

Below is the link to the electronic supplementary material.

Supplementary file1 (DOCX 26 KB)

## Data Availability

No datasets were generated or analysed during the current study.

## Abbreviations

AI: Artificial intelligence
aICU: Average number of patients on ICU who had an ARI as their main diagnosis, per reporting hospital per day
aNA: Average number of newly-admitted patients per reporting hospital per day
ARI: Acute respiratory infection
CPAP: Continuous positive airway pressure
DGKJ: German Society of Pediatrics
DGPI: German Society for Pediatric Infectious Diseases
ICU: Intensive care unit
REDCap: Research Electronic Data Capture
RSV: Respiratory Syncytial Virus
SARS-CoV-2: Severe Acute Respiratory Syndrome Coronavirus 2
VLKKD: German Association of Senior Pediatricians and Pediatric Surgeons
WHO: World Health Organization
