# Supplementary material for: Persistently high burden of acute respiratory infections requiring hospitalization in German pediatric hospitals, fall/winter 2023–2024
Source: Infection. 2025 Mar 11;53(2):717–25. doi: 10.1007/s15010-025-02494-z (PMC11971053; doi:10.1007/s15010-025-02494-z)
Supplement: Supplementary file 1 — Supplementary file1 (DOCX 26 KB) [file 15010_2025_2494_MOESM1_ESM.docx]

**Supplement**

**#the DGPI Ad hoc-ARI study group: all reporting physicians and their affiliations**

Tobias Ankermann 42, Felicitas Anselmino 69, Stefan Arens 33, Sven Armbrust 55, Volker Arpe 20, Elisabeth Bach 46, Gerald Beier 18, Christiane Bell 68, Martin Berghäuser 21, Malte Bergmann 46, Sonja Bernlochner 34, Alexander Bey 20, Anke Beyersdorff 29, Julia Blumör 58, Jasmin Brühler 26, Reinhard Bullmann 11, Reimer Conzelmann 41, Roland Degener 10, Catharina Dobberschütz 37, Svenja Dreßen 1, Thomas Eckardt 18, Torsten Ehrchen 67, Christoph Ehrsam 48, Matthias Endmann 7, Florian Epple 69, Michael Fedlmeier 41, Holger Freymann 21, Marianne Funken 67, Marie Gah 40, Viola Gerstmann 51, Christine Goletz 50, Tatiana Görhardt 58, Katrin Gröger 75, Julia Handermann 40, Madeleine Haug 63, Solvej Heidtmann 49, Maik Heine 37, Matthias Henschen 72, Alexander Herz 47, Mirjam Höfgen 40, Daniel Hubert 6, Birte Hunfeld 52, Conny Huster 76, Katja Hüwe 64, Kristin Jähnert 59, Daniela Jerzyk 28, Petra Kaiser-Labusch 17, Marcus Kania 31, Jens Kästner 39, Margit Kellerer 34, Svetlana Kelzon 67, Chrisoph Kemen 32, Matthias Kettwig 28, Alexander Kiefer 61, Karoline Kinkelin 63, Andreas Klein 43, Christoph Klein 57, Anna-Lisa Kleinmaier 63, Katharina Kliemann 13, Jan Knechtel 73, Felix Knirsch 62, Louise Kobelt 65, Barbara Korinth 14, Ekaterini Kougioumtzi 32, Georgia Koukli 40, Benno Kretzschmar 22, Johannes Kugler 24, Miriam Landauer 58, Alfred Längler 36, Tina Liebscher 1, Vanessa Lionetti 71, Daniela Lubitz 21, Anna-Lena Lubojanski 50, Sa Luo 40, Julia Lutsch 46, Friederike Mattay 44, Anja Mayer 63, Miriam Meusel 56, Ulf Meyer 8, Marko Mohorovicic 68, Johanna Mösler-Tuczek 56, Annika Müller 50, Sven Nipken 38, Ursula Pindur 26, Erika Plattner 61, Simone Pötzsch 60, Lutz Pross 15, Andreas Rößlein 51, Meike Rumpf 19, Jörn Schamell 51, Norbert Schmeja 30, Juliane Schmid 63, Dominik Schneider 2, Josephine Schneider 1, Julia Schönherr 58, Peter Schonhoff 53, Maria Sichardt 10, Daniel Steer 40, Detlef Stein 9, Frank Stemberg 74, Julia Tabatabai 35, Norbert Teig 16, Wolfgang Thomas 70, Renate Turan 14, Vanda Tuxhorn 45, Mirjam Ungerechts 27, Christian v. Schnakenburg 23, Alijda Ingeborg van den Heuvel 54, Noemi Vereb 46, Simone Wagner 3, Sarah Wiehl 63, Christiane Maria Wiethoff 66, Elisabeth Wittig 12, Anne Zeller 63, Ulrich Zügge 25

Department of Pediatrics, University Hospital and Medical Faculty Carl Gustav Carus, Technische Universität Dresden, Dresden, Germany 1, Department of Pediatrics and Adolescent Medicine, Paracelsus Medical University, University Hospital Salzburg, Salzburg, Austria 2, Clinic for Child and Adolescent Medicine, Sana Klinikum Lichtenberg, Academic Teaching Hospital, Charité-Universitätsmedizin Berlin, Germany 3, Clinic of Pediatrics, Municipal Hospital Dortmund, University Witten/Herdecke , Germany 4, Children’s Hospital and Center for Perinatal Medicine, Teaching Hospital of the University of Freiburg, Singen, Germany 5, Aalen, Kinder- und Jugendmedizin, Ostalb-Klinikum 6, Ahlen, Klinik für Kinder und Jugendliche, St. Franziskus-Hospital 7, Annaberg-Buchholz, Klinik für Kinder- und Jugendmedizin, EKA Erzgebirgsklinikum Annaberg gGmbH 8, Arnstadt, Klinik für Kinder-und Jugendmedizin, Ilm-Kreis-Kliniken Arnstadt-Ilmenau gGmbH 9, Bad Hersfeld, Klinik für Kinder- und Jugendmedizin, Klinikum Bad Hersfeld 10, Bad Kreuznach, Kinder- und Jugendmedizin, Diakonie Krankenhaus kreuznacher diakonie 11, Bautzen, Klinik für Kinder- und Jugendmedizin im Krankenhaus Bautzen, Oberlausitz-Kliniken gGmbH 12, Berlin, Kliniken für Kinder- und Jugendmedizin der Charité, Charité Universitätsmedizin Berlin 13, Bernau, Kinder- und Jugendmedizin, Immanuel Klinikum Bernau 14, Böblingen, Klinik für Kinder- und Jugendmedizin, Pädiatrie, Neonatologie, Klinikverbund Südwest Klinikum Sindelfingen-Böblingen 15, Bochum, Kinder- und Jugendmedizin, Universitätsklinikum der Ruhr-Universität Bochum 16, Bremen, Prof.-Hess-Kinderklinik, Klinikum Bremen-Mitte, Gesundheit Nord Klinikverbund Bremen 17, Deggendorf, Kinder- und Jugendmedizin, Neonatologie, Kinderkardiologie, Neuropädiatrie, DonauIsar Klinikum 18, Duisburg, Klinik für Kinder- und Jugendmedizin, Sana Kliniken Duisburg 19, Düren, Kinderklinik, St. Marien Hospital Düren 20, Düsseldorf, Klinik für Kinderheilkunde, Florence Nightingale Krankenhaus der Kaiserswerther Diakonie 21, Eisenach, Klinik für Kinder- und Jugendmedizin Dr. Siegfried Wolff, St. Georg Klinikum Eisenach 22, Esslingen, Klinik für Kinder und Jugendliche, Klinikum Esslingen GmbH 23, Filderstadt, Kinder- und Jugendmedizin, Filderklinik 24, Freiberg, Klinik für Kinder- und Jugendmedizin, Kreiskrankenhaus Freiberg gGmbH 25, Freudenstadt, Kinder- und Jugendmedizin, Krankenhäuser Landkreis Freudenstadt gGmbH 26, Geldern, Klinik für Kinder und Jugendliche, St.-Clemens-Hospital 27, Göttingen, Klinik für Kinder- und Jugendmedizin, Universitätsmedizin Göttingen 28, Greifswald, Kinder- und Jugendmedizin, Universitätsmedizin Greifswald 29, Halle (Saale), Klinik für Kinder- und Jugendmedizin, Krankenhaus St. Elisabeth und St. Barbara 30, Hamburg, Kinder- und Jugendmedizin, Universitätsklinikum Hamburg-Eppendorf 31, Hamburg, Katholisches Kinderkrankenhaus Wilhelmstift 32, Hannover, Auf der Bult Kinder- und Jugendkrankenhaus 33, Hausham, Kinder- und Jugendmedizin, Krankenhaus Agatharied GmbH 34, Heidelberg, Allg.Pädiatrie, Neuropädiatrie, Stoffwechsel, Gastroenterologie, Nephrologie, UniversitätsKlinikum Heidelberg 35, Herdecke, Kinder- und Jugendmedizin, Gemeinschaftskrankenhaus Herdecke gGmbH 36, Hoyerswerda, Kinder- und Jugendmedizin, Lausitzer Seenland Klinikum GmbH 37, Idar-Oberstein, Kinder- und Jugendmedizin/Pädiatrie, Saarland Heilstätten 38, Jena, Klinik für Kinder- und Jugendmedizin, Universitätsklinikum Jena 39, Kaiserslautern, Klinik für Kinder- und Jugendmedizin, Westpfalz Klinikum GmbH 40, Kempten, Kinderheilkunde und Jugendmedizin, Neonatologie, Klinikverbund Kempten-Oberallgäu, Klinikum Kempten 41, Kiel, Klinik für Kinder- und Jugendmedizin, Städtisches Krankenhaus Kiel 42, Köln, Klinik für Kinder- und Jugendmedizin, Kliniken Köln 43, Köln, Kinderklinik, Krankenhaus Porz am Rhein gGmbH 44, Landau, Klinik für Kinder- und Jugendheilkunde, Vinzentius-Krankenhaus Landau 45, Landsberg, Kinder- und Jugendmedizin, Klinikum Landsberg am Lech 46, Lübeck, Klinik für Kinder- und Jugendmedizin, Universitätsklinikum Schleswig-Holstein 47, Meiningen, Kinder- und Jugendheilkunde, Helios Klinikum Meiningen 48, Minden, Universitätsklinik für Kinder- und Jugendmedizin, Johannes Wesling Klinikum Minden 49, Mönchengladbach, Klinik für Kinder und Jugendliche, Städtische Kliniken Mönchengladbach 50, München, Klinik für Kinder- und Jugendmedizin, Klinikum Dritter Orden München-Nymphenburg 51, Münster, Kinder- und Jugendmedizin - Allgemein, St. Franziskus-Hospital GmbH 52, Münster, Kinder- und Jugendmedizin, Clemenshospital 53, Münster, Klinik für Kinder- und Jugendmedizin - Allgemeine Pädiatrie, Universitätsklinikum Münster 54, Neubrandenburg, Klinik für Kinder- und Jugendmedizin, Dietrich-Bonhoeffer-Klinikum 55, Nürnberg, Klinik für Neugeborene, Kinder und Jugendliche, Klinikum Nürnberg Süd 56, Oberhausen, Klinik für Kinder und Jugendliche, St. Clemens-Hospital, Katholisches Klinikum Oberhausen 57, Offenbach, Klinik für Kinder- und Jugendmedizin, Sana Klinikum Offenbach GmbH 58, Perleberg, Klinik für Kinder- und Jugendmedizin, Kreiskrankenhaus Prignitz gGmbH 59, Plauen, Klinik für Kinder- und Jugendmedizin, Helios Vogtland-Klinikum Plauen 60, Regensburg, Klinik und Poliklinik für Kinder- und Jugendmedizin der Universität Regensburg, Barmherzige Brüder Klinik St. Hedwig 61, Remscheid, Klinik für Kinder und Jugendliche, Sana-Klinikum Remscheid 62, Reutlingen, Klinik für Kinder- und Jugendmedizin, Klinikum am Steinenberg, Kreiskliniken Reutlingen GmbH 63, Rheine, Kinderklinik, Klinikum Rheine Mathias-Spital 64, Rodewisch, Klinik für Kinder- und Jugendmedizin, Klinikum Obergöltzsch Rodewisch 65, Rüsselsheim, Klinik für Kinder- und Jugendmedizin, GPR Gesundheits- und Pflegezentrum Rüsselsheim gGmbH 66, Sankt Augustin, Allgemeine Kinder- und Jugendmedizin, Asklepios Kinderklinik 67, Speyer, Klinik für Kinder- und Jugendmedizin, Diakonissen-Stiftungs-Krankenhaus Speyer 68, Starnberg, Klinik für Kinder- und Jugendmedizin, Klinikum Starnberg 69, Trier, Kinder- und Jugendmedizin, Klinikum Mutterhaus der Borromäerinnen gGmbH 70, Velbert, Klinik für Kinder- und Jugendmedizin, Helios Klinikum Niederberg 71, Villingen-Schwenningen, Klinik für Kinderheilkunde, Jugendmedizin und Kinderchirurgie / Sozialpädiatrisches Zentrum, Schwarzwald-Baar Klinikum 72, Witten, Kinder- und Jugendklink, Marien Hospital Witten 73, Worms, Klinik für Kinder- und Jugendmedizin, Klinikum Worms gGmbH 74, Wurzen, Kinder- und Jugendmedizin, Muldentalkliniken Krankenhaus Wurzen 75, Zwickau, Klinik für Kinder- und Jugendmedizin, Heinrich-Braun-Klinikum gGmbH 76
